# HIV status, knowledge and prevention of cervical cancer amongst adolescent girls and women: a secondary data analysis

**DOI:** 10.11604/pamj.2022.41.262.32615

**Published:** 2022-03-31

**Authors:** Godfrey Musuka, Zindoga Mukandavire, Grant Murewanhema, Diego Cuadros, Farirai Mutenherwa, Innocent Chingombe, Rouzeh Eghtessadi, Helena Herrera, Tafadzwa Dzinamarira, Munyaradzi Paul Mapingure

**Affiliations:** 1 Columbia University, Harare, Zimbabwe,; 2 Emirates Aviation University, Centre for Data Science and Artificial Intelligence, Dubai, UAE,; 3 Unit of Obstetrics and Gynecology, Faculty of Medicine and Health Sciences, University of Zimbabwe, Harare, Zimbabwe,; 4 Department of Geography and Geographic Information Science, University of Cincinnati, Cincinnati, USA,; 5 Biomedical Research and Training Institute, Harare, Zimbabwe,; 6 SAfAIDS, Harare, Zimbabwe,; 7 Portsmouth University, Portsmouth, Hampshire, England,; 8 School of Health Systems and Public Health, University of Pretoria, Pretoria, 0002, South Africa

**Keywords:** Cervical cancer, knowledge, factors associated with screening, HIV, Zimbabwe

## Abstract

**Introduction:**

the objective of this manuscript was to describe the knowledge profiles and determinants of cervical cancer screening among HIV positive and negative adolescent girls and women in Zimbabwe.

**Methods:**

we conducted secondary statistical data analysis to explore the determinants of cervical cancer screening among HIV positive and negative adolescent girls and women using Zimbabwe Demographic Health survey for 2015-16.

**Results:**

a total of 9054 adolescent girls aged 15-19, and women aged 20-49 were included in the analysis and the majority (63%) of them resided in rural areas. More than two-thirds (65.9%) had attained secondary level of education. The majority (41.3%) of the adolescent girls and women belonged to the Apostolic sect. A number of key determinants have been identified for being ever screened for cervical cancer. The odds of being ever being screened increased by age, OR(CI) 4.38 (3.22-5.94), p<0.001 for women who are 40 years and older when compared to adolescent and young woman who are between 15-24 years.

**Conclusion:**

our study reports significant programmatic gaps in the provision of cervical cancer screening and treatment services in the country. The nascent Zimbabwe cervical cancer screening and treatment progamme will benefit from expansion of the number of facilities offering the services and the provision of more efficient health education to adolescent women and girls.

## Introduction

Cervical cancer is the fourth most common cancer among women globally and remains a serious global health concern. In 2018 alone, approximately 570 000 incident new cases were diagnosed, and an estimated 311 000 women died from cervical cancer globally [1]. The bulk of the burden is carried in low-resource settings, with Sub-Saharan Africa accounting for the majority of cases [1]. According to the Zimbabwe National Cancer Registry statistics, it is the leading cancer among women in the country and constituted an estimated 33.2% of the cancer burden in this population, with 1308 cases in 2016 [2]. It is now known that high-risk Human Papilloma Viruses (HPV) cause more than 99% of all cervical cases globally, and the development of the cancer goes through well recognised precancerous stages, which develop over a period spanning several years to decades [3]. HPV vaccination provides primary opportunities for prevention and is now considered the most critical intervention for reducing the burden of precancer, and ultimately, invasive cervical malignancy. However, secondary prevention through screening for early detection and treatment of precancerous disease remains the crux of prevention in Zimbabwe and other resource-limited settings as HPV vaccination programmes are still in their infancy [2].

Human Immunodeficiency Virus (HIV) infection is an established risk factor for developing invasive cervical cancer [4]. Zimbabwe has a high prevalence of HIV. According to a recently concluded 2020 national survey, prevalence of HIV among adults in Zimbabwe was 12.9%, which corresponded to approximately 1,225,000 adults living with HIV [5]. The survey reaffirmed gender disparities with an HIV prevalence of 15.3% among women compared to 10.2% among men [5]. Due to the increased risk in this group, WHO guidelines for screening and treatment of precancerous lesions for cervical cancer prevention recommends yearly cervical cancer screening among women living with HIV [6]. Further, the Zimbabwe Ministry of Health and Child Care (MOHCC) is targeting 80% coverage of cancer screening among HIV positive women per year.

Despite a comprehensive cervical cancer screening strategy, HIV positive women still present with advanced disease, requiring specialised treatment which is expensive and not easily accessible for the majority of the population, as Zimbabwe struggles with surgical and radiotherapy capacity, which are confined to the country´s major cities of Harare and Bulawayo [1]. More needs to be done to scale up cervical cancer screening uptake among women living with HIV, premised on understanding and addressing factors that might facilitate and impair this. Thus, this study aimed to describe the knowledge, profiles and determinants of HIV positive and negative women screened for cervical cancer in Zimbabwe using 2015-16 Demographic Health Survey (ZDHS) data.

## Methods

**Study area and data sources:** subjects were enrolled in the ZDHS via a two-stage sampling procedure to select households. A total of 400 ZDHS sample locations were included. There are 9,955 woman respondents in the ZDHS data. This analysis was limited to 9054 (91%) adolescent girls, aged 15-19, and women aged 20 to 49 years who responded to questions on cervical cancer and were also tested for HIV. A trained interviewer administered the questionnaire to participants in either English, or the two major languages that are spoken in Zimbabwe: Shona and Ndebele. Anonymous HIV testing was performed with the informed consent of all sampled individuals. HIV serostatus was determined by testing with the enzyme-linked immunosorbent assay (ELISA) Vironostika Uniform 2 Ag/AB. All those individuals who tested positive were retested with a follow-up ELISA, the Enzygnost® HIV Integral II assay (Siemens). The samples that tested positive to the two tests were classified as HIV positive. When the first and second tests were discordant, a confirmatory test, the HIV 2.2 western blot (DiaSorin), was then used to confirm status [7].

**Statistical analysis:** STATA Version 15.1, Texas USA, was used to conduct statistical analysis for this cross-sectional study. The analysis used simple proportions to describe the characteristics of the adolescent girls and women included in the analysis. We used a simple chi square test to explore associations between categorial variables and ever hearing about cervical cancer. We did the same for the binary outcome variable for being ever screened for cervical cancer. Furthermore, we calculated odds ratio for factors associated with being screening for cervical cancer and then used logistic regression to find the most significant factors after controlling for confounders. In the logistic, we included all factors which were collected in the DHS. In all analysis, statistical significance was set at p = 0.05.

**Ethics approval and consent to participate:** procedures and questionnaires for standard Demographic Health Surveys (DHS) have been reviewed and approved by the International Coaching Federation ICF International Institutional Review Board (IRB). Additionally, country-specific DHS survey protocols are reviewed by the ICF IRB and typically by an IRB in the host country. The ICF International IRB ensures that the survey complies with the U.S. Department of Health and Human Services regulations for the protection of human subjects, while the host country IRB ensures that the survey complies with laws and norms of the nation. In the original primary data collection for each DHS, informed consent was sought from all participants prior to serological testing for HIV. We sought and were granted permission to use the core dataset for this analysis by MEASURE DHS. Informed consent was sought in writing. For minors, consent was obtained from their parents or guardians.

## Results

**Participant demographics:** a total of 9054 adolescent girls and women were included in the analysis and the majority (63%) of them resided in rural areas. 58.4% of the women were married, and more than two thirds had attained at least secondary level of education. The majority (41.3%) of the women belonged to the Apostolic sect. Table 1 shows baseline characteristics of female ZDHS 2015 used in the analysis.

**Table 1:** baseline characteristics of female ZDHS 2015 used in the analysis

Variable	Frequency
**Age group in years**	
15-19	1978 (22.2)
20-24	1633 (17.1)
25-29	1511 (16.7)
30-34	1441 (16.2)
35-39	1085 (12.3)
40-44	882 (9.8)
45-49	524 (5.7)
**Type of residence**	
Urban	3980 (37.0)
Rural	5074 (63.0)
Highest education level	
None	95 (1.3)
Primary	2203 (26.0)
Secondary	6056 (65.9)
Higher	700 (6.8)
**Marital status**	
Never in Union	2437 (25.4)
Married	5170 (58.4)
Living with partner	285 (3.1)
Widowed	388 (4.3)
Divorced	444 (5.1)
Separated	330 (3.6)
**Religion**	
None	435 (5.0)
Traditional	58 (0.7)
Roman Catholic	600 (6.6)
Protestant	1456 (15.7)
Pentecostal	2454 (25.6)
Apostolic sect	3458 (41.3)
Other Christian	561 (4.8)
Muslim	24 (0.3)
Other	8 (0.1)
**HIV status**	
Negative	7471 (83.3)
Positive	1583 (16.7)

**Profiles of women who were ever or never screened for cervical cancer:** Table 2 shows the profiles of adolescent girls and women who had heard about cervical cancer, or never heard about it at all. The proportion of individuals who had heard about cervical cancer increases with age. This proportion was also higher for urban inhabitants compared to those residing in rural areas. The proportion was lowest among those who have never been married or in any union. The proportion was 75% or lower for traditional, Apostolic sect, other Christian sects, no religion and other religions. The proportion increased with increasing wealth quintile. The proportion was higher for the HIV positive women compared to the HIV negative women.

**Table 2:** profiles of women whoever or never heard about cervical cancer

Variable	Ever heard about cervical cancer	Never heard about cervical cancer	P-Value
**Age group in years**		
15-24	2399 (66.6)	1212 (33.4)	
25-39	3538 (87.0)	499 (13.0)	
40+	1262 (88.9)	144 (11.1)	<0.001
**Type of residence**		
Urban	3501 (89.1)	479 (10.9)	
Rural	3698 (73.5)	1376 (26.5)	<0.001
**Highest education level**			
None	56 (58.6)	39 (41.4)	
Primary	1495 (67.8)	708 (32.2)	
Secondary	4963 (82.3)	1093 (17.7)	
Higher	685 (97.5)	15 (2.5)	<0.001
**Marital status**			
Never in Union	1574 (64.4)	863 (35.6)	
Married	4401 (84.2)	769 (15.8)	
Living with partner	240 (84.1)	45 (15.9)	
Widowed	338 (86.6)	50 (13.4)	
Divorced	382 (85.7)	62 (14.3)	
Separated	264 (81.4)	66 (18.6)	<0.001
**Religion**			
None	313 (73.1)	122 (26.9)	
Traditional	43 (73.9)	15 (26.1)	
Roman Catholic	505 (82.8)	95 (17.2)	
Protestant	1216 (82.9)	240 (17.1)	
Pentecostal	2060 (84.5)	394 (15.5)	
Apostolic sect	2625 (75.6)	833 (24.4)	
Other Christian	411 (73.8)	150 (26.3)	
Muslim	21 (82.6)	3 (17.4)	
Other	5 (53.4)	3 (46.6)	<0.001
**Socio-economic status**			
Poorest	909 (66.3)	493 (33.7)	
Poor	970 (73.2)	383 (26.8)	
Middle	1109 (76.0)	349 (24.0)	
Richer	1980 (85.6)	346 (14.4)	
Richest	2231 (89.5)	284 (10.5)	<0.001
**HIV status**			
Negative	5881 (78.4)	1590 (21.6)	
Positive	1318 (83.3)	265 (16.7)	<0.001

**Determinants of cervical cancer screening using proportion, univariate and multivariate analysis:** Table 3 shows data on women who were ever or never screened for cervical cancer. The proportion of being ever screened for cervical cancer increased with increasing age and was higher among the 25-39 and 40+ compared to those 15-19, unadjusted odds ratio (uOR) (95 % confidence interval) (95% CI), 5.22 (4.13-6.60) and 7.54 (5.83-9.77) respectively. It was higher for people residing in urban areas compared to those residing in rural areas, our (95% CI) 2.73 (2.34-3.19). It increased with higher levels of education when considering primary, secondary and higher education attainment with our (95% CI) of 2.32 (1.01-5.35) for those with higher education compared to those with no education. Likewise, it was lowest among those who were never been married or in any union. It was least among the traditional religion followed by the Apostolic sect. It was highest among the richest wealth quantile and was higher for women who are HIV positive, compared to those who are HIV negative, see the rest of our (95% CI) in Table 3.

**Table 3:** profiles of women who were ever or never screened for cervical cancer

Variable	Screened for cervical cancer	Not screened for cervical cancer	Univariate unadjusted Odds Ratio (95% confidence intervals)	P–Value	Multivariate adjusted odds ratio	Variable
**Age group in years**						
15-24	128 (4.5)	2271 (95.6)	1		1	
25-39	733 (19.6)	2805 (80.4)	5.22 (4.13-6.60)	<0.001	2.89 (2.20-3.81)	<0.001
40+	343 (26.0)	919 (74.0)	7.54 (5.83-9.77)	<0.001	4.38 (3.22-5.94)	<0.001
**Type of residence**						
Rural	398 (10.1)	3300 (89.9)	1		1	
Urban	806 (23.5)	2695 (76.5)	2.73 (2.34-3.19)	<0.001	1.39 (1.04-1.85)	0.027
**Highest education level**						
None	8 (16.9)	48 (83.1)	1		1	
Primary	168 (11.3)	1327 (88.7)	0.63 (0.27-1.44)	0.271	0.64 (0.25-1.61)	0.342
Secondary	803 (15.1)	4160 (84.9)	0.88 (0.39-1.98)	0.749	0.71 (0.28-1.77)	0.458
Higher	225 (32.0)	460 (68.0)	2.32 (1.01-5.35)	0.048	1.14 (0.44-2.94)	0.789
**Marital status**						
Never in Union	75 (3.5)	1499 (96.5)	1		1	
Married	884 (18.8)	3517 (81.2)	6.34 (4.68-8.58)	<0.001	4.81 (3.36-6.89)	<0.001
Living with partner	37 (11.7)	203 (88.3)	3.63 (2.18-6.05)	<0.001	3.58 (1.99-6.45)	<0.001
Widowed	82 (22.3)	256 (77.7)	7.82 (5.18-11.79)	<0.001	3.82 (2.33-6.28)	<0.001
Divorced	72 (18.8)	310 (81.2)	6.30 (4.13-9.61)	<0.001	3.73 (2.34-5.94)	<0.001
Separated	54 (21.1)	210 (78.9)	7.28 (4.62-11.48)	<0.001	5.19 (3.16-8.55)	<0.001
**Religion**						
None	42 (14.3)	271 (85.7)	1		1	
Traditional	4 (6.5)	39 (93.5)	0.42 (0.13-1.36)	0.149	0.34 (0.10-1.20)	0.093
Roman Catholic	103 (19.2)	402 (80.8)	1.43 (0.90-2.25)	0.129	1.10 (0.68-1.78)	0.702
Protestant	254 (19.3)	962 (80.7)	1.44 (0.95-2.18)	0.086	1.15 (0.75-1.79)	0.520
Pentecostal	389 (18.3)	1671 (81.7)	1.35 (0.90-2.01)	0.148	1.01 (0.66-1.54)	0.970
Apostolic sect	324 (11.6)	2301 (88.4)	0.79 (0.53-1.18)	0.247	0.86 (0.57-1.29)	0.469
Other Christian	82 (18.8)	329 (81.2)	1.39 (0.86-2.24)	0.175	1.28 (0.78-2.13)	0.331
Muslim	5 (23.5)	16 (76.5)	1.84 (0.59-5.73)	0.291	1.74 (0.54-5.59)	0.353
Other	1 (42.9)	4 (57.1)	4.51 (0.46-44.02)	0.195	1.94 (0.21-18.35)	0.562
**SES**						
Poorest	75 (8.3)	834 (91.7)	1		1	
Poor	78 (8.0)	892 (92.0)	0.96 (0.67-1.36)	0.801	0.99 (0.69-1.41)	0.948
Middle	117 (9.1)	992 (90.9)	1.10 (0.79-1.53)	0.573	1.06 (0.76-1.49)	0.716
Richer	378 (19.4)	1602 (80.6)	2.66 (1.99-3.57)	<0.001	2.17 (1.52-3.11)	<0.000
Richest	556 (25.1)	1675 (74.9)	3.69 (2.79-4.89)	<0.001	3.00 (1.97-4.57)	<0.000
**HIV status**						
Negative	893 (13.9)	4988 (86.1)	1		1	
Positive	311 (24.0)	1007 (76.0)	1.95 (1.63-2.32)	<0.001	1.58 (1.30-1.93)	<0.001

**Figure 1:**
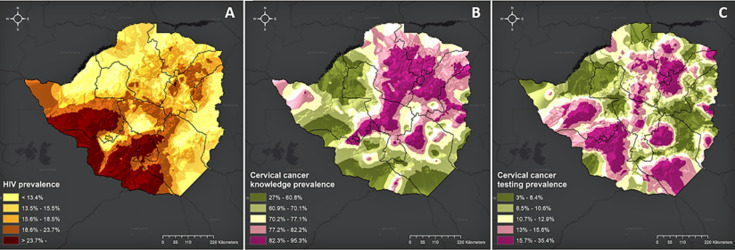
A) HIV in females; B) prevalence of knowledge about cervical cancer; and C) prevalence of cervical cancer testing

From multivariate analysis., we identified the key adjusted determinants forever being screened for cervical cancer. The adjusted odds ratio (aOR) (95% CI) of being screened increased with age and were 4.38 (3.22-5.94) for women who are 40 years and older when compared to those who are between 15-24 years. Women residing in urban areas were more likely to be screened for cervical cancer, compared to their rural counterparts aOR (95% CI) 1.39 (1.04-1.85). There was no significant effect of educational status on ever being screened for cervical cancer. Compared to those who were never in any union, there was a significant association between cervical cancer screening and all the other marital unions. Women in the top wealth quantile were more likely to be screened compared to women in the lowest wealth quantile. HIV-positive women were more likely to be screened compared to HIV-negative women, see the rest of aOR (95% CI) figures in Table 3. Figure 1 shows maps for HIV in females, prevalence of knowledge about cervical cancer, and prevalence of cervical cancer testing. It is very interesting to see that knowledge of cervical cancer has an opposite distribution than HIV prevalence.

## Discussion

The ZDHS did not include HPV vaccination data, which is the major intervention for cervical cancer prevention in adolescent girls. Data on the uptake of HPV vaccines is critical for programmatic implementation and planning. Nevertheless, regardless of this caveat, the study provided important insights regarding cervical cancer screening in Zimbabwe. Targeted screening for HIV-positive women is expected to improve uptake in this important population. In Zimbabwe, screening for HIV negative women through visual inspection of the cervix procedures or cytology ideally commences at the age of 25; however, there are recommendations to screen HIV positive women for cervical cancer even in adolescents. Consensus guidelines clearly stating the position would be useful for dealing with policy and practice inconsistencies and variations which can threaten programme success.

Despite a good proportion of women having heard about cervical cancer and screening, women still do access this service. Several individual-related barriers, community-related and health system-related factors can serve as barriers to screening uptake, despite some level of awareness. In a literature review by Chidyaonga-Maseko and colleagues, age, marital status, socioeconomic status, cultural and religious beliefs of the women were noted to be some of the individual-related barriers [8]. In this study, across all age groups, marital groups, educational levels, religious groups and socioeconomic statuses, the proportions of unscreened women were higher than those of screened women. However, the proportion of HIV positive women screened for cervical cancer was higher compared to HIV negative women. Women who are HIV positive may be exposed to more awareness campaigns and calls for screening as they visit health facilities regularly for supplies of their HIV medicines, alongside information via support groups. Though the difference was statistically significant, the numbers screened in the HIV positive cohort were far to low for what is desired target number. Despite regular visits to clinics for refill of antiretroviral medicines, there are still significant gaps and missed opportunities for cervical cancer screening.

Primary prevention through vaccination for high-risk HPVs is likely to become the key prevention intervention for cervical cancer in the future, as vaccination coverage becomes more widespread [9]. However, secondary prevention through screening leading to early detection and treatment of precancerous disease remains an important strategy in the country. Countries that have comprehensive cervical cancer screening programs have seen a marked reduction in the incidence of invasive cancer, attributable to this secondary prevention [9]. More frequent, yearly screening is recommended for HIV-infected women, whose risk of developing cervical cancer is significantly augmented. In a review by Stelzle and others, the pooled risk of cervical cancer was increased in HIV positive women (RR 6.07, 95% CI 4.40-8.37) [4]. Furthermore, in Southern Africa, 63.8% (95% CI 58.9-68.1) of women with cervical cancer were HIV-infected. It is for this reason there is need to have robust cervical cancer screening programs actively targeting HIV positive women, especially those of low socio-economic status and with-out easy access to healthcare.

Visual inspection of the cervix with acetic acid cervicography (VIAC) and conventional cytology through Pap smears are the widely available screening modalities in Zimbabwe, especially in urban settings. VIAC programs are supported by development partners, and are generally available free of charge; however, limitations in resources, infrastructure, manpower and patient knowledge play a role in reduced uptake of these preventative services [10]. Their successful uptake is critical for reducing the incidence of invasive cervical cancer [11]. Unfortunately, this was identified as insufficient in this study, which is consistent with findings from other studies carried out in resource-limited settings. Additionally, the current COVID-19 scenario involving lockdowns, fear of exposure to infection, and other barriers, fewer women will be routinely accessing cervical cancer screening programmes [12].

If the gap in screening is to be effectively addressed, stakeholders and policymakers in public health must aim to critically understand the barriers to uptake of screening. This study presents data on the knowledge profiles and determinants of cervical cancer screening uptake among a sample population of women living with HIV in Zimbabwe. Knowledge about cervical cancer and the existence of screening programmes is a critical determinant of the uptake of screening. However, logistical barriers such as transport requirements and distance to nearest health facility offering screening services can be additional barriers to accessing services [13]. Rural Zimbabwe has poor road and digital networks, and the cost of transport can be high, particularly for an unemployed population. A hyperinflationary economic environment may also lead to prioritization of services to address existing health issues over uptake of health promotion services [14].

Health policymakers and those involved in strategic planning must urgently implement a multi-pronged community mobilization to raise awareness and improve health seeking behaviour, especially amongst the Apostolic sects, which have been shown in other studies to have poor health seeking behaviour [15]. Our study showed extensive local variation in awareness and knowledge of cervical cancer, and prevalence of cervical cancer testing, and in HIV disease burden across Zimbabwe. The high-resolution maps generated by this study have identified areas wherein high density of HIV-infected individuals with low awareness and knowledge of cervical cancer. This information is key for geographical prioritization of interventions by the Zimbabwe MOHCC. These results suggest that there is need to tailor intervention programmes to target and address specific local needs, including gaps in knowledge and limited access, using a micro-planning approach to programming which has been described in literature.

## Conclusion

Cervical cancer screening uptake is critical for reducing the incidence of cervical cancer in Zimbabwe. This is particularly important among women living with HIV/AIDS, whose risk of developing invasive cervical cancer is significantly augmented. A multi-sector, multi-stakeholder approach involving different players in public health is required to urgently address the determinants of this uptake, especially as health services have been significantly affected by the ongoing COVID-19 pandemic. As restoration of essential services begins, cancer screening programmes must be given priority.

### What is known about this topic

- *Cervical cancer is the leading gynaecological malignancy in Zimbabwe;*
- *Primary prevention through HPV vaccination for adolescent girls is the most critical intervention to reduce the incidence of cervical cancer;*
- *Secondary prevention through screening remains an important intervention in Zimbabwe, where HPV vaccination is still in its infancy*.

### What this study adds

- *There are still significant gaps in the cervical cancer screening programme in Zimbabwe;*
- *The proportion of individuals who had heard about cervical cancer increases with age. This proportion was also higher for urban inhabitants compared to those residing in rural areas. The proportion was lowest among those who have never been married or in any union;*
- *The odds of being ever screened increased by age, OR(CI) 4.38 (3.22-5.94), p<0.001 for adolescent girls and women who are 40 years and older when compared to adolescent and young women, those who are between 15-24 years*.

## References

[1] World Health Organization (WHO) Cervical Cancer: Overview, Eliminating cervical cancer.

[2] Gabaza C, Chonzi P, Chadambuka A, Shambira G, Juru TP, Gombe NT (2019). Utilization and outcomes of cervical cancer screening services in Harare City, 2012-2016: a secondary data analysis. BMC Health Serv Res.

[3] World Health Organization (WHO) Cervical cancer.

[4] Stelzle D, Tanaka LF, Lee KK, Khalil AI, Baussano I, Shah AS, McAllister DA, Gottlieb SL, Klug SJ, Winkler AS, Bray F (2021). Estimates of the global burden of cervical cancer associated with HIV. Lancet Glob Health.

[5] PHIA Project (2020). Zimbabwe Summary Sheet.

[6] WHO (2013) Guidelines for screening and treatment of precancerous lesions for cervical cancer prevention: supplemental material: GRADE evidence-to-recommendation tables and evidence profiles for each recommendation.

[7] Zimbabwe National Statistics Agency and ICF International (2016) Zimbabwe Demographic and Health Survey 2015: Final Report.

[8] Chidyaonga-Maseko F, Chirwa ML, Muula AS (2015). Underutilization of cervical cancer prevention services in low and middle income countries: a review of contributing factors. Pan Afr Med J.

[9] Arbyn M, Weiderpass E, Bruni L, de Sanjosé S, Saraiya M, Ferlay J, Bray F (2020). Estimates of incidence and mortality of cervical cancer in 2018: a worldwide analysis. Lancet Glob Health.

[10] Kuguyo O, Matimba A, Tsikai N, Magwali T, Madziyire M, Gidiri M, Dandara C, Nhachi C (2017). Cervical cancer in Zimbabwe: a situation analysis. Pan Afr Med J.

[11] Seay JS, Kobetz E (2018). Optimizing cervical cancer screening and triage in low-resource settings. J Glob Oncol.

[12] Murewanhema G (2021). Reduced cervical cancer screening in Zimbabwe as an indirect impact of the COVID-19 pandemic: implications for prevention. Pan Afr Med J.

[13] Nyamambi E, Murendo C, Sibanda N, Mazinyane S (2020). Knowledge, attitudes and barriers of cervical cancer screening among women in Chegutu rural district of Zimbabwe. Cogent Social Sciences.

[14] Salatin P, Bridari M (2014). Effects of inflation on women´s health in selected middle-income countries. African Journal of Health Economics.

[15] Mapingure M, Mukandavire Z, Chingombe I, Cuadros D, Mutenherwa F, Mugurungi O, Musuka G (2021). Understanding HIV and associated risk factors among religious groups in Zimbabwe. BMC Public Health.

